# F33: A-: B-, IncHI2/ST3, and IncI1/ST71 plasmids drive the dissemination of *fosA3* and *bla*_CTX−M−55/−14/−65_ in *Escherichia coli* from chickens in China

**DOI:** 10.3389/fmicb.2014.00688

**Published:** 2014-12-16

**Authors:** Xiaoyun Yang, Wuling Liu, Yiyun Liu, Jing Wang, Luchao Lv, Xiaojie Chen, Dandan He, Tong Yang, Jianxia Hou, Yinjuan Tan, Li Xing, Zhenling Zeng, Jian-Hua Liu

**Affiliations:** Laboratory of Veterinary Pharmacology, College of Veterinary Medicine, South China Agricultural UniversityGuangzhou, China

**Keywords:** *Escherichia coli*, poultry, fosfomycin, plasmids, animal reservoirs, CTX-M

## Abstract

The purpose of this study was to examine the occurrence of fosfomycin-resistant *Escherichia coli* from chickens and to characterize the plasmids carrying *fosA3*. A total of 661 *E. coli* isolates of chicken origin collected from 2009 to 2011 were screened for plasmid-mediated fosfomycin resistance determinants by PCR. Plasmids were characterized using PCR-based replicon typing, plasmid multilocus sequence typing, and restriction fragment length polymorphisms. Associated addiction systems and resistance genes were identified by PCR. PCR-mapping was used for analysis of the genetic context of *fosA3*. Fosfomycin resistance was detected in 58 isolates that also carried the *fosA3* gene. Fifty-seven, 17, and 52 FosA3-producers also harbored *bla*_CTX−M_, *rmtB*, and *floR* genes, respectively. Most of the 58 *fosA3*-carrying isolates were clonally unrelated, and all *fosA3* genes were located on plasmids belonged to F33:A-:B- (*n* = 18), IncN-F33:A-:B- (*n* = 7), IncHI2/ST3 (*n* = 10), IncI1/ST71 (*n* = 3), IncI1/ST108 (*n* = 3), and others. The genetic structures, IS*26-ISEcp1*-*bla*_CTX−M−55_-*orf477*-*bla*_TEM-1_-IS*26*-*fosA3*-1758bp-IS*26* and IS*Ecp1*-*bla*_CTX−M−65_-IS*903*-iroN-IS*26*-*fosA3*-536bp-IS*26* were located on highly similar F33:A-:B- plasmids. In addition, *bla*_CTX−M−14_-*fosA3*-IS*26* was frequently present on similar IncHI2/ST3 plasmids. IncFII plasmids had a significantly higher frequency of addiction systems (mean 3.5) than other plasmids. Our results showed a surprisingly high prevalence of *fosA3* gene in *E. coli* isolates recovered from chicken in China. The spread of *fosA3* can be attributed to horizontal dissemination of several epidemic plasmids, especially F33:A-:B- plasmids. Since coselection by other antimicrobials is the major driving force for the diffusion of the *fosA3* gene, a strict antibiotic use policy is urgently needed in China.

## Introduction

The increasing occurrence of bacterial infections caused by multidrug resistant gram-negative *Enterobacteriaceae* (MDR-GNB) has reignited interest in the old antibiotic, fosfomycin (Falagas et al., 2010). In recent years, fosfomycin has been widely recommended for treating uncomplicated urinary tract infection (UTI) due to its ease of administration and powerful bactericidal activity against MDR-GNB, especially extended-spectrum β-lactamase (ESBL)-producing and fluoroquinolone-resistant *Escherichia coli* (Falagas et al., 2010; Gardiner et al., 2014; Karlowsky et al., 2014). At the same time, occasional reports of fosfomycin-resistant *E. coli* strains have emerged (Lee et al., 2012; Ho et al., 2013a; Lai et al., 2014). Mutations in chromosomal genes are the main mechanism for fosfomycin resistance in *E. coli* (Michalopoulos et al., 2011). However, the transferable fosfomycin resistance genes, *fosA*, *fosC2*, and *fosK*, were recently identified in Asian countries (Wachino et al., 2010; Hou et al., 2012, 2013; Lee et al., 2012; Ho et al., 2013a,b; Kitanaka et al., 2014). Although fosfomycin use in animals is prohibited in China, a high prevalence of the plasmid-mediated fosfomycin-resistance gene *fosA3* was observed in *E. coli* isolates from pet in China (Hou et al., 2012). We also detected *fosA3* in *E. coli* isolates from food animal recovered from 2004 to 2008, but with a relatively lower prevalence compared with pet isolates (Hou et al., 2013). *fosA3* is always co-transferred with *bla*_CTX−M_ genes and the dissemination of *fosA3* among pet isolates may be attributed to co-selection by cephalosporins (Hou et al., 2012, 2013). The frequency of *bla*_CTX−M_ in *E. coli* isolated from food animals in China has generally been reported to be low prior to 2008, but has increased in prevalence in recent years, especially in chicken isolates (Liu et al., 2007; Li et al., 2010; Zheng et al., 2012). To determine if the occurrence of fosfomycin resistance has also increased with the increasing frequency of *bla*_CTX−M_ in food animal isolates in recent years, we screened *E. coli* isolates of chicken origin collected during 2009–2011 for fosfomycin resistance and plasmid-mediated fosfomycin resistance genes. Characterization of *fosA3*-encoding plasmids as well as the association of *fosA3* with other resistance genes, such as *bla*_CTX−M_, was also examined.

## Materials and methods

### Bacterial isolates

A total of 661 *E. coli* isolates were collected from healthy or diseased chickens in China from 2009 to 2011. Two hundred and ten *E. coli* from sick chickens at 57 farms were recovered from clinical samples submitted to diagnostic laboratories in Guangdong, Anhui, and Shandong province. The remaining 451 isolates were obtained from fecal samples of healthy chickens from 33 chicken farms located in different geographic regions of China, including Jiangsu, Henan, Guangxi, Gansu, and Guangdong provinces. Sample collection, culture, and *E. coli* isolation were performed as described previously (Liu et al., 2007; Zheng et al., 2012). *E. coli* isolates were identified by standard biochemical tests. Assumed fosfomycin- resistant *E. coli* isolates were selected using Mueller–Hinton (MH) agar plates supplemented with 128 mg/L fosfomycin (Northeast Pharmaceutical Group, Ltd) and 25 μg/mL glucose-6-phosphate and subjected to further study.

### Antimicrobial susceptibility testing

Antimicrobial susceptibility test was performed by the agar dilution method on Mueller–Hinton agar plates. The antimicrobial drugs tested included cefotaxime, fosfomycin, gentamicin, amikacin, florfenicol, tetracycline, ciprofloxacin, colistin, and imipenem. Moreover, the isolates were investigated for resistance to tigecycline (15 μg), and piperacillin/tazobactam (110 μg) by the agar disk diffusion method. Both susceptibility tests were carried out and evaluated according to the protocols recommended in VET01-S2 and M100-S23 of the Clinical and Laboratory Standards Institute (2013a,b). *E. coli* ATCC 25922 was used as the control strain.

### Detection of antimicrobial resistance genes

The assumed fosfomycin resistant *E. coli* isolates were screened for the presence of the plasmid-mediated fosfomycin resistance genes *fosA3*, *fosC2*, and *fosA* by PCR amplification using primers described previously (Ho et al., 2013b). PCR results were confirmed by sequencing. The *fosA3*-positive strains were also evaluated for the presence of *bla*_CTX−M_, *rmtB*, and *floR* genes by PCR (Chen et al., 2004, 2007; Sun et al., 2010). The genotype of *bla*_CTX−M_ was confirmed by PCR and DNA sequencing.

### Strain typing

Pulse-field gel electrophoresis (PFGE) of *Xba*I digested genomic DNA was carried out as described previously (Gautom, 1997) using the CHEF-MAPPER System (Bio-Rad Laboratories, CA, USA). PFGE patterns were analyzed with BioMumerics software (Applied Maths) and were interpreted according to the well-established criteria described by Tenover (Tenover et al., 1995). Isolates that had PFGE patterns with no more than six different bands were considered clonally related. Isolates were further analyzed by multilocus sequence typing (MLST), which was performed according to the specifications given at http://mlst.warwick.ac.uk. MLST profiles were analyzed by Bionumerics.

### Conjugation experiments and plasmid analysis

The transferability of *fosA3* genes was investigated by conjugation experiments using streptomycin-resistant *E. coli* C600 as the recipient strain. Transconjugants were selected on MacConkey agar plates supplemented with fosfomycin (200 μg/mL) and streptomycin (2000 μg/mL). Transfer frequencies were calculated as the number of transconjugants per recipient. Transformation experiments were performed to obtain a single plasmid carrying *fosA3*, as verified by S1 nuclease PFGE when more than one plasmid was co-transferred. The antimicrobial susceptibility of the transconjugants/transformants was determined by the agar dilution method, and the presence of *fosA3*, *bla*_CTX−M_, *rmtB*, and *floR* in the transconjugants/transformants was confirmed by PCR. PCR-based replicon typing (PBRT) was performed on all transconjugants/transformants carrying a single plasmid, as described by Carattoli et al. (2005). To better characterize IncFII, IncI, and IncHI2 plasmids, replicon sequence typing (RST), plasmid multilocus sequence typing (pMLST), and plasmid double locus sequence typing (pDLST) were performed according to the procedure described previously (Garcia-Fernandez et al., 2008; Garcia-Fernandez and Carattoli, 2010; Villa et al., 2010), and alleles were assigned by submitting the amplicon sequence to the plasmid MLST database (www.pubmlst.org/plasmid/). F33:A-:B-, IncI1, and IncHI2 plasmids carrying *fosA3* were extracted by a rapid alkaline lysis procedure (Takahashi and Nagano, 1984) and further compared using restriction fragment length polymorphism (RFLP) analysis with *EcoR*I restriction enzymes.

### Analysis of the genetic environment of the *fosA3* gene

The genetic context surrounding the *fosA3* gene was investigated by PCR mapping and sequencing. The primers used to determine the regions upstream and downstream of the *fosA3* genes are listed in Table S1.

### Nucleotide sequence accession numbers

The new surrounding region of the *fosA3* gene found in this study has been deposited in the GenBank database under the following accession numbers: KJ668701 and KJ668702.

## Results

### Prevalence of plasmid-mediated fosfomycin resistance genes

Of the 661 *E. coli* isolates from chicken examined, 58 (8.8%) showed resistance to fosfomycin and carried the *fosA3* gene. These 58 isolates were recovered from chickens in 37 different farms located in five provinces (Guangdong, Anhui, Shandong, Guangxi, and Henan).

### Characterization of *fosA3*-carrying *E. coli* isolates

PFGE was successfully performed on 56 *E. coli* isolates carrying *fosA3*, and 52 different *Xba*I PFGE patterns were identified (Table 1). Forty-four different sequence types (STs) were detected among 58 *fosA3*-positive isolates, including 16 new STs (Table 1 and Supplementary Figure S1). Isolates belonging to ST48 (ST10 clonal complex) were detected in 6 isolates from 6 farms. Minimum spanning tree of MLST of 58 *fosA3*-carrying *E. coli* isolates by Bionumerics.

**Table 1:** **Characterization of *fosA3*-carrying *E. coli* isolates**.

**Isolate^a^**	**Farm^b^**	**Isolation date**	**PFGE pattern^c^**	**MLST**	**Resistance profile^d^**
AHC8	F1	2011.6	1	ST4472	AMK, CTX, GEN, CIP, FFC, TET
AHC9	F1	2011.6	2	ST48	CTX, GEN, CIP, TET
AHC12	F1	2011.6	3	ST533	CTX, GEN, CIP, FFC, TET
AHC16	F2	2011.6	3	ST533	CTX, GEN, CIP, FFC, TET
AHC19	F4	2011.6	3	ST533	CTX, GEN, CIP, FFC, TET
AHC14	F1	2011.6	4	ST746	CTX, CIP, FFC, TET
AHC17	F2	2011.6	5	ST4483	AMK, CTX, GEN, CIP, FFC, TET
AHC18	F3	2011.6	6	ST4447	CTX, CIP, TET
AHC23	F5	2011.6	7	ST2607	AMK, CTX, GEN, CIP, FFC, TET
AHC24	F5	2011.6	8	ST155	AMK, CTX, GEN, CIP, FFC, TET
AHC26	F6	2011.6	9	ST23	AMK, CTX, GEN, CIP, TET
AHC27	F6	2011.6	10	ST2179	CTX, GEN, CIP, FFC, TET
AHC30	F6	2011.6	11	ST226	CTX, CIP, FFC, TET
AHC33	F7	2011.6	12	ST101	AMK, CTX, GEN, CIP, FFC, TET
AHC51	F9	2011.7	13	ST57	CTX, GEN, CIP, FFC, TET
AHC52	F9	2011.7	14	ST206	AMK, CTX, GEN, CIP, FFC, TET
AHC76	F8	2011.7	15	ST4466	AMK, CTX, GEN, CIP, FFC, TET
AHC54	F9	2011.7	16	ST155	AMK, CTX, GEN, CIP, TET
AHC55	F9	2011.7	17	ST162	CTX, GEN, CIP, FFC, TET
AHC57	F10	2011.7	18	ST10	AMK, CTX, GEN, FFC, TET
AHC60	F11	2011.7	19	ST48	AMK, GEN, CIP, FFC, TET
AHC66	F12	2011.7	20	ST48	CTX, GEN, CIP, FFC, TET
AHC67	F12	2011.7	21	ST2223	CTX, GEN, FFC, TET
AHC69	F13	2011.7	22	ST2847	CTX, CIP, FFC, TET
AHC72	F13	2011.7	23	ST2847	CTX, CIP, FFC, TET
AHC80	F14	2011.7	24	ST155	AMK, CTX, GEN, CIP, FFC, TET
GDC27	F15	2010.8	25	ST156	CTX, GEN, FFC, TET
GDC40	F16	2010.8	26	ST744	CTX, GEN, CIP, FFC, TET
GDC54	F18	2010.8	27b	ST2496	CTX, GEN, CIP, FFC, TET
GDC46	F17	2010.8	27a	ST4460	CTX, GEN, CIP, FFC, TET
GDC47	F17	2010.8	28	ST746	CTX, CIP, FFC, TET
GDC114^*^	F28	2010.9	29	ST48	AMK, CTX, GEN, CIP, FFC, TET
GDC56	F19	2010.8	30a	ST359	CTX, GEN, CIP, FFC, TET
GDC58	F23	2010.8	30b	ST4461	CTX, CIP, FFC, TET
GDC61	F20	2010.8	31	ST4473	CTX, GEN, FFC, TET
GDC15	F21	2010.8	32	ST4465	CTX, CIP, FFC
GDC16	F21	2010.8	33	ST4474	AMK, CTX, GEN, CIP, FFC, TET
GDC17	F21	2010.8	34	ST1518	CTX, CIP, FFC, TET
GDC24	F15	2010.8	35	ST4477	CTX, CIP, FFC, TET
GDC37	F22	2010.8	36	ST4459	CTX, GEN, FFC, TET
GXC03^*^	F24	2009.7	37	ST2847	CTX, GEN, CIP, FFC, TET
GXC19^*^	F25	2009.7	38	ST4360	CTX, GEN, CIP, FFC, TET
HNC02^*^	F31	2009.7	39	ST4464	AMK, CTX, GEN, FFC, TET
HNC06^*^	F31	2009.7	40	ST779	CTX, TET
SDC20	F35	2009.6	41	ST48	CTX, FFC, TET
SDC10	F32	2009.6	42	ST4498	AMK, CTX, GEN, CIP, FFC, TET
SDC12	F32	2009.6	43	ST4497	AMK, CTX, GEN, CIP, FFC, TET
SDC04	F33	2009.5	44	ST602	CTX, CIP, FFC, TET
SDC01	F34	2009.5	45	ST4462	AMK, CTX, GEN, CIP, FFC, TET
SDC11	F34	2009.6	46	ST602	AMK, CTX, GEN, CIP, TET
SDC15	F36	2009.5	47	ST219	AMK, CTX, GEN, CIP, FFC, TET
GD326	F27	2011.3	48	ST1589	CTX, CIP, FFC, TET
GDC1-4^*^	F28	2010.9	49	ST453	CTX, GEN, CIP, FFC, TET
GDC240^*^	F29	2010.8	50	ST354	AMK, CTX, GEN, CIP, FFC, TET
GDC540^*^	F29	2010.8	51	ST48	CTX, GEN, FFC, TET
GDC1-2^*^	F28	2010.9	52	ST93	CTX, GEN, CIP, FFC, TET
GDC127^*^	F30	2010.9	smeared	ST156	CTX, GEN, CIP, FFC, TET
SDC13	F37	2009.5	smeared	ST398	CTX, GEN, CIP, FFC, TET

a *Different provinces are indicated as follows: AH, Anhui; GD, Guangdong; GX, Guangxi; HN, Henan; SD, Shandong. Isolates from which the fosA3 gene can be transferred to the recipient by conjugation are underlined. Healthy animals are indicated by an asterisk*.

b *F1 to F36, farm 1 to farm 36, respectively*.

c *PFGE types (1, 2, 3, etc.) were assigned by visual inspection of the macrorestriction profile. Patterns that differed by fewer than six bands were considered to represent subtypes within the main group (30a, 30b, etc.). NT, nontypeable*.

d *AMK, amikacin; CTX, cefotaxime; CIP, ciprofloxacin; FFC, florfenicol; GEN, gentamicin; TET, tetracycline. The antimicrobial susceptibility results were interpreted according to breakpoint of CLSI (M100-S23), except that florfenicol (≥32 μg/mL) was interpreted according to breakpoint of European Committee on Antimicrobial Susceptibility Testing (EUCAST). All isolates were susceptible to colistin, imipenem, piperacillin-tazobactam, and tigecycline. Resistance phenotypes transferred to the recipient by conjugation ortransformationare underlined*.

Of the 58 FosA3-producing *E. coli* isolates examined in this study, 58 (100%), 57 (98.3%), 57 (98.3%), 52 (89.7%), 49 (84.5%), 44 (75.9%), and 21 (36.2%) were resistant to ampicillin, cefotaxime, tetracycline, florfenicol, ciprofloxacin, gentamicin, and amikacin, respectively (Table 1). All isolates were susceptible to colistin, imipenem, piperacillin-tazobactam, and tigecycline.

Results of screening for resistance genes showed that 57 of the 58 FosA3-producing *E. coli* isolates carried *bla*_CTX−M_ genes, including *bla*_CTX−M−55_ (*n* = 24), *bla*_CTX−M−65_ (*n* = 20), *bla*_CTX−M−14_ (*n* = 11), *bla*_CTX−M−123_ (*n* = 3), *bla*_CTX−M−3_ (*n* = 2), *bla*_CTX−M−64_ (*n* = 1), and *bla*_CTX−M−15_(*n* = 1). Five isolates carried two different *bla*_CTX−M_ genes. In addition, 17 and 52 isolates harbored *rmtB* and *floR* genes, respectively.

### Analysis of *fosA3* plasmids

*fosA3* genes from 50 isolates were successfully transferred by conjugation. The *fosA3*-bearing plasmids in the remaining eight isolates and six transconjugants carrying multiple plasmids were transferred by transformation. Three transformants carried multiple plasmids and were not studied further. The 55 *fosA3* plasmids ranged in size from 45 to 230 kb and contained IncFII (*n* = 29), IncI1 (*n* = 9), IncHI2 (*n* = 12), and IncN (*n* = 4) replicons (Table 2). In addition, seven plasmids were fused plasmids, which contained both IncN and IncFII replicons. The replicon type for one plasmid could not be determined by the PBRT method. Interestingly, the majority of IncFII plasmids associated with the *fosA3* gene were classified as F33:A-:B- by RST. Subtyping of IncI1 plasmids revealed three sequence types, including ST71 (*n* = 3), ST108 (*n* = 3), and a new sequence type ST136 (*n* = 2). The IncI1 plasmid from isolate AHC60 was not typable because the *trbA* and *pilL* alleles were not detected. Ten IncHI2 plasmids were assigned to ST3 by pDLST, while the other two IncHI2 plasmids were not typable due to failure to detect the smr0199 loci. By restriction analysis of plasmid DNA using *EcoR*I, 21 F33:A-:B- plasmids, eight IncI1 plasmids, and nine IncHI2 plasmids were divided into 10, eight, and nine groups, respectively, which exhibited small band differences. The conjugation frequencies of F33:A-:B- and IncN-F33:A-:B-plasmids were 10^−6^ to 10^−8^, while IncI1 and IncHI2 plasmids were 10^−5^ to 10^−6^.

**Table 2:** **Characterization of some plasmids carrying *fosA3***.

**Plasmid(s)**	**Co-transfer of other resistance gene(s)**	**Context of *fosA3*^a^**	**Plasmid**			
			**Size (kb)**	**Replicon type**	***Eco*RI RFLP ^b^**	**Addiction systems**
GDC24, GDC58, AHC18, SDC13, GDC40, GDC47, GDC1-4, GDC114, SDC04	*bla*_CTX−M−55_	I	~75	F33:A-:B-	A1	*hok-sok*, *pemKI*, *srnBC*
AHC33, GDC240	*bla*_CTX−M−65_, *rmtB*	III	~75	F33:A-:B-	A1	*hok-sok*, *pemKI*, *srnBC*
AHC17, AHC26	*bla*_CTX−M−55_, *rmtB*	I	~100	N-F33:A-:B-	A2	*hok-sok*, *pemKI*, *srnBC*, *vagCD*
AHC24	*bla*_CTX−M−55_*, rmtB*, *floR*	I	~110	N-F33:A-:B-	A3	*hok-sok*, *pemKI*, *srnBC*, *vagCD*
GDC54	*bla*_CTX−M−65_	III	~75	F33:A-:B-	A4	*hok-sok*, *pemKI*, *srnBC*, *vagCD*
AHC23	*bla*_CTX−M−65_, *rmtB*	III	~80	F33:A-:B-	A5	*hok-sok*, *pemKI*, *srnBC*
GDC46	*bla*_CTX−M−55_	III	~75	F33:A-:B-	A6	*hok-sok*, *pemKI*, *srnBC*, *vagCD*
SDC01	*bla*_CTX−M−55_, *rmtB*	I	~100	N-F33:A-:B-	A7	*hok-sok*, *pemKI*, *srnBC*, *vagCD*
AHC76	*bla*_CTX−M−55_	I	~75	F33:A-:B-	A8	*hok-sok*, *pemKI*, *srnBC*
HNC02	*bla*_CTX−M−65_, *rmtB*	III	~80	F33:A-:B-	A9	*hok-sok*, *pemKI*, *srnBC*, *vagCD*
AHC52	*bla*_CTX−M−55_, *rmtB*, *floR*	I	~110	N-F33:A-:B-	A10	*hok-sok*, *pemKI*, *srnBC*, *vagCD*
GDC17	*bla*_CTX−M−55_, *floR*	I	~95	N-F33:A-:B-	B	*hok-sok*, *pemKI*, *srnBC*, *vagCD*
AHC9	*bla*_CTX−M−55_	I	~65	N-F33:A-:B-	C	*pemKI*, *srnBC*, *vagCD*
AHC69, AHC72	*bla*_CTX−M−65_	I	~75	F33:A-:B-	ND	*hok-sok*, *pemKI*, *srnBC*, *vagCD*
AHC27	*bla*_CTX−M−65_	I	~115	I1/ST71	D1	*pemKI*, *pndCA*, *vagCD*
GDC27	*bla*_CTX−M−65_, *floR*	I	~125	I1/ST71	D2	*pemKI*
AHC30	*bla*_CTX−M−55_, *floR*	I	~105	I1/ST136	D3	*hok-sok*, *pemKI*, *pndCA*, *vagCD*
GXC19	*bla*_CTX−M−65_, *floR*	II	~125	I1/ST71	D4	*pndCA*
AHC54	*bla*_CTX−M−123_	I	~110	I1/ST108	D5	*pemKI*, *pndCA*, *vagCD*
AHC14	*bla*_CTX−M−123_	I	~115	I1/ST108	D6	*pndCA*
AHC55	*bla*_CTX−M−123_	I	~110	I1/ST108	D7	*hok-sok*, *pemKI*, *pndCA*
SDC11	*bla*_CTX−M−14_, *rmtB*	V	~100	I1/ST136	D8	*pemKI*, *pndCA*
AHC60	*rmtB*	Unknown	~120	I1	E	*pndCA*, *vagCD*
AHC66, AHC67	*bla*_CTX−M−65_, *floR*	I	~230	HI2/ST3	F1	none
AHC57	*bla*_CTX−M−14_, *floR*	IV	~230	HI2/ST3	F2	none
AHC80	*bla*_CTX−M−14_, *rmtB*, *floR*	IV	~230	HI2/ST3	ND	*vagCD*
GDC540	*bla*_CTX−M−14_, *floR*	V	~230	HI2/ST3	F3	*pemKI*, *vagCD*
GDC15	*bla*_CTX−M−65_, *floR*	III	~230	HI2/ST3	F4	none
GDC61	*bla*_CTX−M−14_, *floR*	IV	~230	HI2	F5	*pemKI*, *vagCD*
GXC03	*bla*_CTX−M−15_	Unknown	~230	HI2/ST3	F6	*vagCD*
GDC127	*bla*_CTX−M−14_, *floR*	V	~230	HI2/ST3	F7	none
HNC06	*bla*_CTX−M−14_	V	~230	HI2/ST3	F8	*vagCD*
GD326	*bla*_CTX−M−14_, *floR*	V	~230	HI2	F9	none
SDC20	*bla*_CTX−M−65_, *floR*	I	~230	HI2/ST3	ND	none
SDC10, SDC12	*rmtB*, *floR*	I	~45	N	ND	none
SDC15	*rmtB*	I	~50	N	ND	*vagCD*
AHC51	*bla*_CTX−M−65_	I	~50	N	ND	none
GDC1-2	*bla*_CTX−M−3_, *floR*	I	~75	F2:A-:B-	ND	*hok-sok*, *pemKI*, *vagCD*
GDC56	*bla*_CTX−M−3_	I	~75	F14:A-:B-	ND	*hok-sok*, *pemKI*, *vagCD*
AHC12	*bla*_CTX−M−65_, *floR*	I	~80	F18:A-:B-	ND	*pemKI*, *srnBC*, *vagCD*, *ccdAB*
AHC16	*bla*_CTX−M−14_, *floR*	I	~80	F18:A-:B-	ND	*pemKI*, *srnBC*, *ccdAB*
GDC16		I	~70	unknown	ND	*vagCD*

a *Contexts of fosA3 were as follows: I, IS26-316bp-fosA3-1758bp-IS26; II, IS26-316bp-fosA3-536bp-IS26; III, bla_CTX−M−14_-1135bp-fosA3-1758bp-IS26; IV, bla_CTX−M−14_-611bp-fosA3-1222bp-IS26*.

b *Restriction fragment length polymorphism (RFLP) patterns differed by only a few bands (n = 1–3) were assigned to the same RFLP profile. ND, not determined*.

Co-transfer of resistance to other antimicrobials (cefotaxime, aminoglycosides, florfenicol, and tetracycline) was observed in 54 of the 55 transconjugants/transformants harboring a single plasmid. *bla*_CTX−M_, *rmtB*, and *floR* genes were co-transferred with *fosA3* to the recipients from 50, 14, and 19 donors, respectively.

### Addiction systems of *fosA3* plasmids

The 55 *fosA3* plasmids carried 0-4 addiction systems (mean 2.4). Six different systems, namely *ccdAB*, *hok-sok*, *pemKI*, *pndAC*, *snrBC*, and *vagCD* systems were detected. The most frequently represented systems were *pemKI*, followed by *vagCD*, *hok-sok*, and *srnBC* (Table 2). All F33: A-: B- plasmids except one (AHC9) had *pemKI*, *hok-sok*, and *srnBC*. The average number of addiction systems detected was the highest (3.5) among IncFII plasmids, followed by IncI1 plasmids (2.2), which were significantly higher than the remaining plasmids (*P* < 0.01). IncN and IncHI2 plasmids were mostly devoid of the addiction systems tested in the study (Table 2).

### Genetic environment of *fosA3*

**Figure 1:**
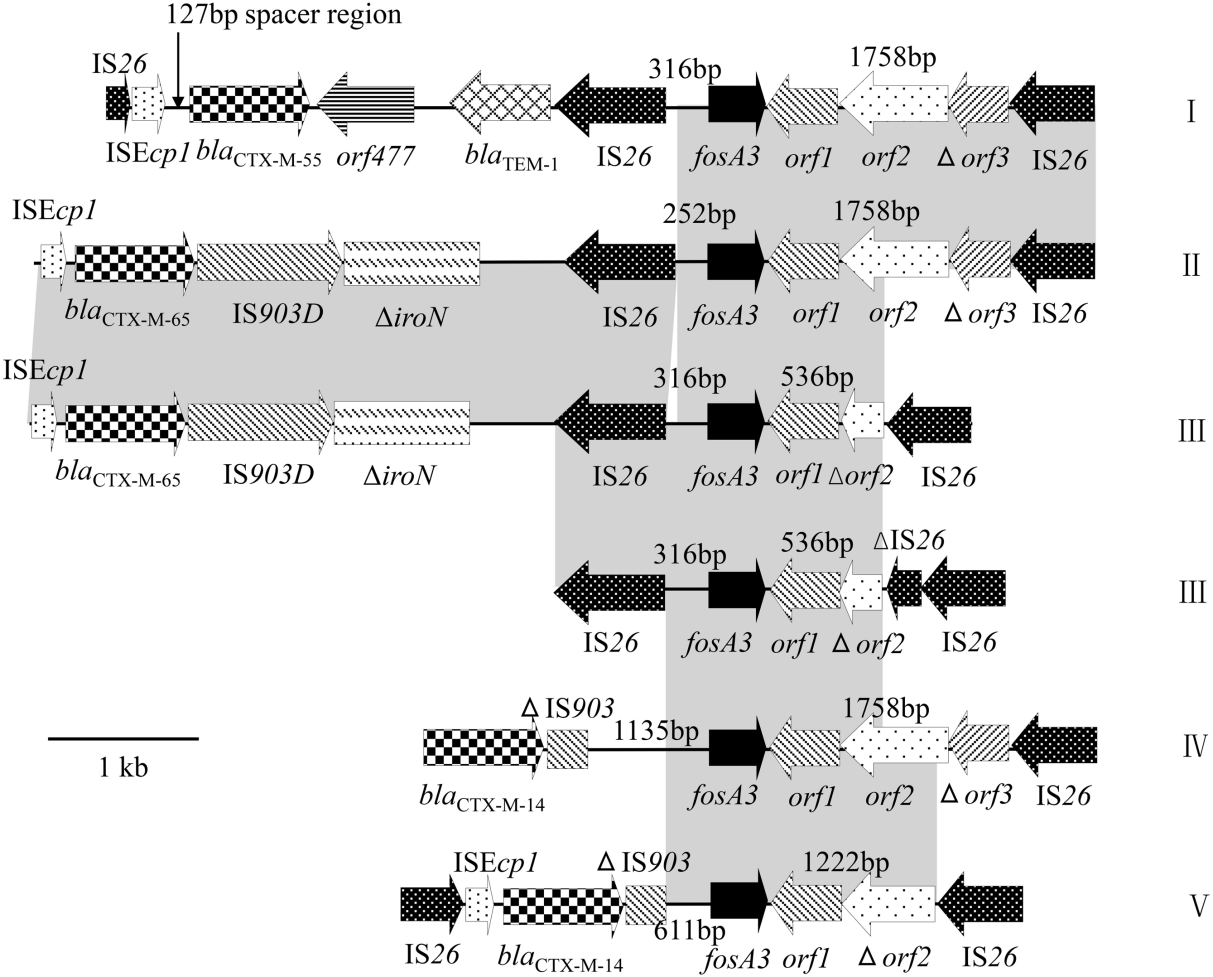
**Schematic representation of the genetic contexts of *fosA3* gene**. Regions of 100% homology are shaded in gray.

The regions surrounding *fosA3* were determined by PCR mapping and sequencing. Different genetic contexts of *fosA3* were designated as types I–V (Table 2 and Figure 1). An IS*26* element was found to be located downstream of *fosA3* in all the isolates except AHC60, AHC8, and GXC03. In these three isolates, the genetic elements downstream of *fosA3* could not be defined. The sizes of the spacer regions between the 3′ end of *fosA3* and IS*26* varied (1758, 536, and 1222 bp; Table 1). Upstream of *fosA3*, four different genetic organizations were identified. In 48 isolates, IS*26* was located 316 bp upstream of *fosA3* (type I, III), and in 1 isolate (GXC19), IS*26* was located 252 bp upstream of *fosA3* (type II). In nine isolates, *bla*_CTX−M−14_ was identified 1135 bp upstream of *fosA3* (type IV) or 611 bp upstream of *fosA3* (type V). The type V structure (*bla*_CTX−M−14_-611bp-*fosA3*-1222bp-IS*26*) was 100% identical to that found in the plasmids of ECO021TF (accession no. JQ343849, human *E. coli*, Korea, 2009), pHP48 (AB778503, human *E. coli*, Japan, 2010), and pN0863T (JQ823170, dog *E. coli*, Hong Kong, 2008). The type IV structure (*bla*_CTX−M−14_-1135bp-*fosA3*-1758bp-IS*26*) was 100% identical to that found in the plasmid of EC0121TF (accession no. JX442753, chicken *E. coli*, 2009) from Hong Kong and was found to be located on IncHI2 plasmids. IncHI2 was also found to be associated with type V structures. However, type I and type III structures were usually found to be associated with F33:A-:B- and IncI1 plasmids. In 15 F33:A-:B- or N-F33:A-:B- plasmids, a structure comprising IS*26*, truncated IS*Ecp1*, *bla*_CTX−M−55_, *orf477*, and a truncated *bla*_TEM−1_, was found upstream of the type I structure. This genetic environment surrounding the *fosA3* gene was 100% in sequence identity to the plasmid carried by *E. coli* HP558 (AB778291, human *E. coli*, 2010) from Japan. Also, in five F33:A-:B- plasmids and four IncI1 plasmids, a structure comprising IS*26*, truncated IS*Ecp1*, *bla*_CTX−M−65_, IS*903*, and *iroN* was found upstream of the type I structure and type III structure, respectively. The genetic structure (IS*Ecp1*-*bla*_CTX−M−65_-IS*903*-*iroN*-IS*26*-316bp-*fosA3*-536bp-IS*26*) located on F33:A-:B- plasmids was almost identical to those found in plasmids pXZ (JF927996, duck *E. coli*, 2008) and pHN7A8 (JN232517, dog *E. coli*, 2008) from China. The genetic structure (IS*Ecp1*-*bla*_CTX−M−65_-IS*903*-iroN-IS*26*-252bp-*fosA3*-1758bp-IS*26*) carried by IncI1 plasmids represented a novel genetic environment and was first identified in this study. In one isolate with the type III *fosA3* context, the IS*26* downstream of *fosA3* was truncated by another IS*26*.

## Discussion

In this study, we investigated the prevalence of fosfomycin resistance in *E. coli* isolated from chickens from 2009 to 2011 in China. Our previous study showed that fosfomycin resistance was unusual among *E. coli* isolates from food animals during the period from 2004 to 2008 (Hou et al., 2013). However, in this study, fosfomycin resistance was detected in 8.8% of *E. coli* isolates from chickens. This frequency was significantly higher than that reported in other countries (Karageorgopoulos et al., 2012; Karlowsky et al., 2014), and also higher than that in isolates from humans in China (Lai et al., 2014). Fosfomycin is not approved for use in food animals in China. Thus, the high frequency of fosfomycin resistance found in this study was unexpected and was not due to the selective pressure resulting from exposure to fosfomycin. Coselection by other antimicrobials, especially third-generation cephalosporins, seemed to play a major role in facilitating the spread of this plasmid-mediated fosfomycin-resistance determinant since almost all *fosA3* plasmids also carried *bla*_CTX−M_ genes. In addition, the intensive use of florfenicol and gentamicin might also have favored the dissemination of *fosA3* in the chicken population as florfenicol- and gentamicin-resistance genes were usually co-transferred with *fosA3*. Other studies also observed the cotransfer of *bla*_CTX−M_ genes with the *fosA3* gene and the cotransfer of chloramphenicol resistance (Hou et al., 2012, 2013; Ho et al., 2013a; Sato et al., 2013; Lai et al., 2014).

Most of the *fosA3*-positive isolates (48/58) found in this study were recovered from diseased chickens. Generally, chicken farmers will use antimicrobial agents to control diseases; if such disease controls fail, they will send the diseased animals to diagnostic laboratories for diagnosis and treatment. Thus, the diseased chickens have most likely been subjected to more serious antimicrobial selective forces than healthy chickens before they are sent to diagnostic laboratories, suggesting that other antimicrobials may be the driving force for the observed increase in fosfomycin resistance and may affect the dissemination of *fosA3* in chickens. In support of this, we surveyed the antimicrobial usage history of 15 farms (data not shown) and found that cephalosporins and florfenicol were frequently used in most chicken farms.

The spread of the *fosA3* gene among *E. coli* isolates of chicken origin was not attributed to clonal transfer of FosA3-producers, but was instead caused by several epidemic plasmids, including F33:A-:B-, IncI1/ST108, IncI1/ST71, and IncHI2/ST3 plasmids, which have been disseminated in multiple chicken farms found in different geographic regions of China.

The combination of *fosA3* and F33:A-:B- plasmids has been frequently identified in several Asia countries (Hou et al., 2012, 2013; Lee et al., 2012; Sato et al., 2013; Pan et al., 2014). In our previous study on isolates from pets and food animals collected during the period from 2004 to 2008, *fosA3* genes usually co-existed with *ba*_CTX−M−65_ on F33:A-:B- plasmids (Hou et al., 2012, 2013). However, in this study, *fosA3* genes frequently co-existed with *bla*_CTX−M−55_ on a similar or identical genetic structure carried by F33:A-:B- plasmids, similar to that of the two F33:A-:B- plasmids found in Japan and Korea (Lee et al., 2012; Sato et al., 2013). These data indicated that F33:A-:B- plasmids carrying identical or similar mobile multiresistance regions have disseminated in animals and humans in different Asian countries. Therefore, these plasmids are capable of spreading very efficiently and may be the major vehicle contributing to the spread of the *fosA3* gene. The successful dissemination of F33:A-:B- plasmids may be attributed to the presence of addiction systems (*pemKI*, *hok-sok*, *srnBC*), which ensure the stable maintenance of the plasmid during cell division (Hou et al., 2012; He et al., 2013b). Though the F33:A-:B- plasmid has only been identified in Asian countries, it may be possible for the plasmid to disseminate worldwide by international travel or animal and food trade.

IncI1 plasmids carrying *fosA3* have previously been reported in China and Japan (Hou et al., 2013; Sato et al., 2013). In this study, three different IncI1 pMLST plasmid types were associated with *fosA3*, namely ST71, ST108, and ST136. Three ST71 plasmids from different provinces (Anhui, Guangxi, and Guangdong) carried both *fosA3* and *bla*_CTX−M−65_. Interestingly, ST71 carrying *bla*_CTX−M−14_ and *fosA3* was also detected in isolates from humans in Japan (Sato et al., 2013). ST108, first reported in our previous study as a *bla*_CTX−M−123_ carrier (He et al., 2013a), also harbored *bla*_CTX−M−123_ in this study. In a pig farm in the United Kingdom, the ST108 IncI1 plasmid carrying the *bla*_CTX−M−1_ gene had disseminated across multiple genera (Freire Martin et al., 2014). IncHI2 plasmids have been found to be associated with ESBL genes in *Enterobacteriaceae*, but were more common in *Salmonella enterica* than in *E. coli* (Garcia-Fernandez and Carattoli, 2010). This study reported the identification of *fosA3* genes on IncHI2 plasmids for the first time. Most *fosA3* genes in IncHI2 plasmids have similar genetic environments, mainly *bla*_CTX−M−14_-*fosA3*-IS*26*. However, this structure was located on the IncN plasmid in one isolate from Korea (Lee et al., 2012). Our findings revealed that the mobile element IS*26* and co-selection with *bla*_CTX−M_ genes played a critical role in the rapid transfer of the *fosA3* gene between diverse epidemic plasmids. Since *fosA3* can be carried by several successfully disseminated plasmids (F33:A-:B-, ST108/ST71 IncI1, and ST3 IncHI2) and CTX-M-type ESBL producers are distributed globally in a variety of settings (Woerther et al., 2013), we should pay close attention to the worldwide dissemination of *fosA3* in the near future. More studies are required to investigate the spread of *fosA3* gene in other countries as well as in other hosts and environments.

In conclusion, our study reported a surprisingly high prevalence of the plasmid-mediated fosfomycin-resistance gene *fosA3* in *E. coli* isolates from chicken in China. Fosfomycin has become one of the limited treatment options for critically ill patients with multidrug-resistant bacteria, especially carbapenem-resistant gram-negative bacteria (Dortet et al., 2014), further spread of the *fosA3* gene would be a serious public health concern. Measures must be implemented to avoid the selection and spread of fosfomycin-resistant strains. Since the *fosA3* gene is usually cotransferred with *bla*_CTX−M−55_, *bla*_CTX−M−65_, *bla*_CTX−M−14_, *floR*, and *rmtB* genes on several epidemic plasmids, reduction in total antimicrobial use, particularly cephalosporins, in food animal production in China may help to control the spread of plasmid-mediated fosfomycin-resistance genes.

### Conflict of interest statement

The authors declare that the research was conducted in the absence of any commercial or financial relationships that could be construed as a potential conflict of interest.
