# Supplementary material for: F33: A-: B-, IncHI2/ST3, and IncI1/ST71 plasmids drive the dissemination of fosA3 and blaCTX−M−55/−14/−65 in Escherichia coli from chickens in China
Source: Front Microbiol. 2014 Dec 16;5:688. doi: 10.3389/fmicb.2014.00688 (PMC4267423; doi:10.3389/fmicb.2014.00688)

**Supplementary material.**

**Figure S1** Minimum spanning tree of multilocus sequence typing (MLST) of fifty-eight *fosA3*-carrying *Escherichia coli* isolates.

Each circle denotes a particular MLST type as is shown. Different colours represent different areas in China that *Escherichia coli* isolates originally come from. The figures on the connection lines denote the numbers of different loci variants between the two MLST types. Thick, solid lines indicate connections between single-locus variants; thin, solid lines indicate double-locus variants; dashed lines indicate types that differ in more than two loci. The white circles denote STs for those no clonal complex has been assigned on the MLST website (http://mlst.ucc.ie/). The shadowing surrounding the various types indicates the groupings obtained by Bionumerics analysis where groups were created if neighbors differed in only one of the seven alleles.


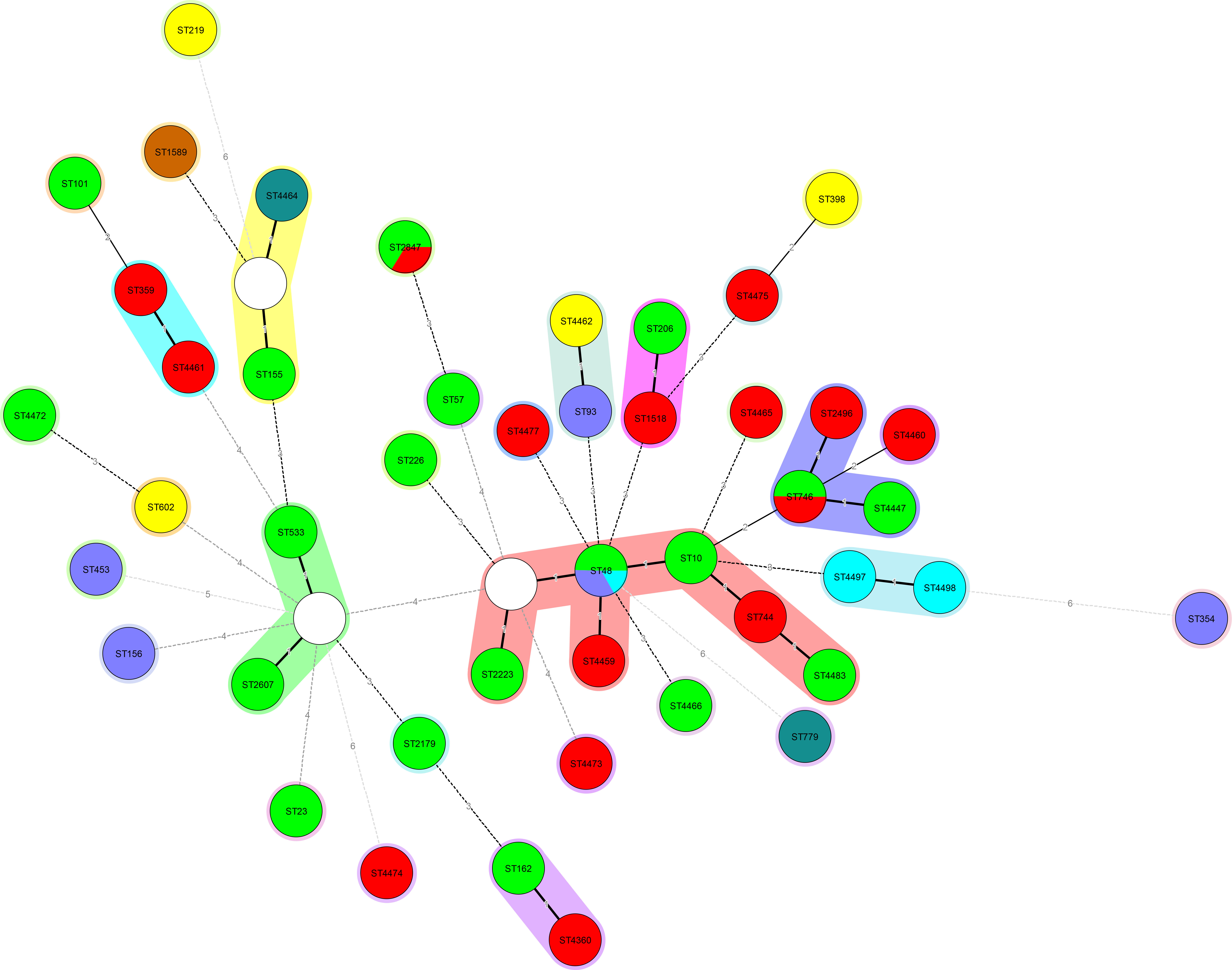

Supplement: Supplementary file 1 [file DataSheet1.DOC]
